# Protective Influence of SGLT-2 Inhibitors Against Heart Failure in Type 2 Diabetes Mellitus Through Longitudinal Clinical Database Analysis

**DOI:** 10.3390/jcm13237093

**Published:** 2024-11-24

**Authors:** Attila Csaba Nagy, Ágnes Tóth, Natália Bak, Battamir Ulambayar, Amr Sayed Ghanem, Ferenc Sztanek

**Affiliations:** 1 Department of Health Informatics, Faculty of Health Sciences, University of Debrecen, 4032 Debrecen, Hungary; baknatalia44@mailbox.unideb.hu (N.B.); ulambayar.battamir@etk.unideb.hu (B.U.); aghanem@etk.unideb.hu (A.S.G.); 2 Department of Integrative Health Sciences, Faculty of Health Sciences, University of Debrecen, 4028 Debrecen, Hungary; toth.agnes@etk.unideb.hu; 3 Division of Metabolism, Department of Internal Medicine, Faculty of Medicine, University of Debrecen, 4032 Debrecen, Hungary; sztanek@belklinika.com

**Keywords:** diabetes mellitus, cardiovascular disease, heart failure, SGLT-2 inhibitors, clinical laboratory tests

## Abstract

**Background**: Sodium–glucose co-transporter 2 (SGLT-2) inhibitors, initially designed for type 2 diabetes, promote glucose excretion and lower blood glucose. Newer analogs like empagliflozin and dapagliflozin improve cardiovascular outcomes through mechanisms other than glycemic control, including blood pressure reduction and anti-inflammatory effects. Given the high cardiovascular risk present in diabetes, our study aims to emphasize the cardioprotective benefits of SGLT-2 inhibitors as a preventive therapy for heart failure (HF) in high-risk T2DM patients. **Methods**: Using data from 2542 patients identified by the ICD-10 E11 code from 2016 to 2020, this longitudinal study excluded those with E10 codes or those undergoing insulin treatment to focus on non-insulin-dependent T2DM. a multiple logistic regression model assessed HF incidence while adjusting for demographics and HbA1c. **Results**: SGLT-2 inhibitor use significantly lowered the odds of heart failure events (OR = 0.55, 95% CI: 0.31–0.99, *p* = 0.046), with a significant difference by gender (OR = 0.45, 95% CI: 0.28–0.71, *p* = 0.001) and eGFR (OR = 0.98, 95% CI: 0.97–0.99, *p* = 0.004). **Conclusions**: The real-world data highlight SGLT-2 inhibitors as promising for HF prevention and broader cardiometabolic health in T2DM, with potential value in managing complex comorbid profiles.

## 1. Introduction

Sodium–glucose co-transporter 2 (SGLT-2) inhibitors are a class of orally administered agents originally designed for the management of type 2 diabetes mellitus (T2DM) [1]. Their mechanism of action involves the inhibition of SGLT-2 channels [2], expressed predominantly in the brush-border membrane of the early segment (S1) of renal proximal tubule [3], playing an important role in the reabsorption of glucose with a large capacity for transporting glucose through the plasma membrane against the concentration gradient [4]. This results in an increase in urinary glucose excretion and, consequently, a decrease in blood glucose levels [1,5].

SGLT-2 inhibitors are derivatives of phlorizin (named originally phloridzin), the natural glucoside, which was first isolated from the bark of an apple tree by Laurent-Guillaume de Koninck, together with Jean Servais Stas, in 1835 [6]. Phlorizin was found to be an inhibitor of SGLT-2 and, to a lesser extent, of SGLT-1 [7,8], and it was also found to have an inhibitory effect on the glucose transporter 1 (GLUT1). The potential for glucose uptake blockage in various tissues prevented its clinical applications [9]. In addition, its poor absorption in the small intestine and its hydrolysability by intestinal lactase also contributed to its pharmaceutical limitations. However, synthetic analogs of phlorizin, involving empagliflozin, canagliflozin, dapagliflozin, ertugliflozin, luseogliflozin, ipragliflozin, sotagliflozin, tofogliflozin, and remogliflozin etabonate [2], were developed with improved stability, bioavailability, and selectivity for SGLT-2 [7,8].

Although SGLT-2 inhibitors have been proposed as a therapeutic strategy for diabetes, they have gained attention for their cardiovascular benefits and uses in the context of obesity as well. Notably, they have been approved as a novel treatment for both the prevention of heart failure (HF) and to reduce cardiovascular morbidity and mortality [10]. Major randomized clinical trials such as EMPA-REG OUTCOME [11] and the CANVAS Program [12] showed that the rate of the primary composite cardiovascular outcome and of death in the case of patients with T2DM at high risk for cardiovascular events was lowered when receiving empagliflozin, and canagliflozin, respectively, compared with a placebo; furthermore, the DAPA-HF Trial [13] displayed shorter hospitalization, a decreased risk of worsening HF and a reduced ejection fraction (HFrEF) in patients who received dapagliflozin compared with those who were given a placebo, regardless of the presence of diabetes. SOLOIST-WHF was the first trial that provided evidence that sotagliflozin significantly reduces primary end-point events (mortality from cardiovascular causes and hospitalization) in T2DM patients with heart failure with a preserved ejection fraction (HFpEF) [14]. Subsequently, two dedicated HFpEF studies, EMPEROR-Preserved [15] and DELIVER [16], showed that empagliflozin and dapagliflozin were also effective in reducing primary end-point events.

In addition to glycaemic control, there are several hypothesized mechanisms behind the effectiveness of SGLT-2 inhibitors in reducing mortality and morbidity associated with HF [17,18] and type 2 diabetes mellitus [19,20]. The diuretic effect of SGLT-2 inhibitors contributes to a reduction in plasma volume, therefore lowering blood pressure [21,22,23], which can alleviate the symptoms of heart failure by decreasing the cardiac workload. This reduction in preload and afterload enhances myocardial efficiency [24,25], which is crucial for patients with HF as it can lead to an improved cardiac output and reduced hospitalization rates [26,27]. Furthermore, weight loss [28] is associated with SGLT-2 inhibitors, which can be attributed to caloric excretion through glucosuria. A reduction in body weight can have significant benefits for cardiovascular health, since obesity is a common comorbidity in T2DM patients that exacerbates cardiovascular risk [29,30]. In addition to these advantages, SGLT-2 inhibitors are thought to exert cardioprotective effects through various biochemical pathways, including the cardiac energy metabolism, as well as enhancing fatty acid oxidation, and leading to a moderate increase in ketone body production [31], which may serve as an alternative energy source for the heart [24,25]. Moreover, SGLT-2 inhibitors have been associated with reductions in oxidative stress and inflammation [32], which are critical factors in the pathophysiology of heart failure [25,33]. These anti-inflammatory effects may also contribute to improved vascular function, further supporting cardiovascular health [34].

Globally, about 422 million people live with diabetes, which might lead to serious complications over time, e.g., damage to the heart [35], which directly impairs cardiac function, while additional comorbidities, such as coronary disease, renal dysfunction, hypertension, obesity, and other metabolic disorders might indirectly effect and strengthen the link between diabetes and heart failure [36]. Thus, the aim of our study was to underline the cardioprotective benefits of SGLT-2 inhibitors using clinical data and to highlight the importance of the preventive treatment of patients with T2DM, who represent a population at high risk for heart failure.

## 2. Materials and Methods

### 2.1. Study Design and Data

Data from the Department of Internal Medicine at the University Hospital of Debrecen in Hungary were used in this longitudinal study, which included data from 2016 to 2020. Information on 2542 people with T2DM who were diagnosed according to the clinical guidelines is included in the dataset. These individuals had never been diagnosed with heart failure prior to study inclusion. The E11 ICD-10 code filter was applied to the hospital database to selectively identify patients with T2DM for the study cohort. Patients were excluded if they had records indicating the E10 code (typically related to type 1 diabetes), had preexisting heart failure at baseline, were below the age of 45, or had an estimated glomerular filtration rate (eGFR) below 30 mL/min/1.73 m^2^, as this condition could independently influence heart failure risk and confound the study’s outcomes. To monitor the health status and clinical features of the patients during follow-up years, the dataset contains demographic information and clinical laboratory parameters that were gathered annually, as well as therapies during this time. The dataset was sorted by patient ID and year as longitudinal data for the statistical analysis.

### 2.2. Variables

Diagnosis codes were extracted from patient records, and the relevant condition indicator was set to 1 for each patient for the year in which an applicable ICD-10 code was found in the diagnosis records. ICD-10 diagnosis codes for diabetic nephropathy, diabetic retinopathy, diabetic neuropathy, atherosclerosis, stroke, acute myocardial infarction, heart failure, and peripheral artery disease were used to characterize clinical outcomes. Binary indicator variables were developed for each condition to indicate whether it existed or not for each patient-year. Incidence of heart failure was the study’s primary outcome. Categorical variables were developed based on whether the laboratory parameters were elevated, lowered, or within the normal range using the normal reference levels used in the Clinical Center of Laboratory Medicine, University of Debrecen [31]. International, generic, and brand names were used to filter and identify medications approved for use in Hungary that belong to the SGLT-2 inhibitor group from the list of treatments for patients with T2DM. A binary variable was then created to indicate whether an SGLT-2 inhibitor treatment was administered.

### 2.3. Statistical Analysis

#### 2.3.1. Baseline Characteristics

To examine outcomes across baseline characteristics, we used Pearson’s chi-squared test. Categorical variables included in the comparison were gender, age group, creatinine levels, LDL levels, HbA1c, triglyceride levels, and the presence of comorbid conditions (e.g., atherosclerosis, stroke, acute myocardial infarction (AMI), peripheral artery disease, nephropathy, retinopathy, and neuropathy).

#### 2.3.2. Multiple Logistic Regression Model

We applied multiple logistic regression models to analyze the association between SGLT-2 inhibitor use and heart failure outcomes, adjusted for time. Logistic regression was chosen over Cox proportional hazards modeling due to the discrete annual structure of the data, which made logistic regression more suitable for estimating the probability of heart failure within each interval. Cox regression, which assumes continuous time-to-event data, was deemed less appropriate for this dataset. Additionally, logistic regression demonstrated greater robustness given the limited number of failure events and did not require the proportional hazards assumption. It also provided easily interpretable odds ratios. Model fit comparisons using AIC and BIC further supported logistic regression as the more parsimonious and reliable approach for this analysis.

#### 2.3.3. Model Performance Evaluation

To evaluate model discrimination, we performed Receiver Operating Characteristic (ROC) analysis, calculating the area under the curve (AUC) as a summary measure. ROC analysis assesses the sensitivity (true positive rate) and specificity (true negative rate) of the model at various probability thresholds, providing insight into its predictive accuracy. The ROC analysis demonstrated good discriminative ability, with an area under the curve (AUC) indicating strong predictive accuracy. All statistical analyses were conducted using Stata IC version 17.0 software [37]. The research involving human participants received approval from the Ethics Committee at the University of Debrecen. All studies were carried out in compliance with relevant local laws and institutional guidelines. The Ethics Committee at the University of Debrecen granted the necessary approval (5610-2020) on 17 December 2020.

## 3. Results

The baseline characteristics of patients with T2DM indicated a median age of 65 years (IQR: 58–71). Gender distribution was slightly female-dominated, with 54.72% of participants being female (*n* = 1391) and 45.28% being male (*n* = 1151). Comorbid conditions were prevalent, including 28.21% (*n* = 717) of patients with atherosclerosis, 25.33% (*n* = 644) with myocardial ischemia, 7.67% (*n* = 195) with a history of stroke, and 15.11% (*n* = 384) with peripheral artery disease. Additional complications included nephropathy in 17.15% (*n* = 436), retinopathy in 14.63% (*n* = 372), and neuropathy in 18.69% (*n* = 475). Median laboratory values were as follows: HbA1c at 7.2% (IQR: 6.5–8.2), LDL cholesterol at 2.76 mmol/L (IQR: 2.05–3.50), HDL cholesterol at 1.20 mmol/L (IQR: 1.00–1.45), and triglycerides at 1.70 mmol/L (IQR: 1.24–2.50) (Table 1).

Pearson’s chi-squared analysis based on baseline characteristics revealed no significant differences in heart failure (HF) incidence by gender (*p* = 0.099), age group (*p* = 0.723), LDL levels (*p* = 0.642), or triglyceride levels (*p* = 0.383). However, HbA1c ≥ 7% was significantly associated with increased HF incidence (*p* = 0.01). Comorbid conditions were found to significantly impact HF incidence: patients with atherosclerosis (*p* = 0.003), AMI (*p* < 0.001), peripheral artery disease (*p* = 0.045), nephropathy (*p* = 0.034), and retinopathy (*p* = 0.036) had worse outcomes. Although neuropathy showed a trend towards significance, it did not reach statistical significance (*p* = 0.066). Stroke was not significantly associated with HF incidence (*p* = 0.186). The use of SGLT-2 inhibitors showed a highly significant association with lower HF incidence (*p* < 0.001) (Table 2).

The multiple logistic regression model identified a significant association between the use of SGLT-2 inhibitors and reduced odds of HF incidence (OR = 0.55, 95% CI: 0.31–0.99, *p* = 0.046). Additionally, female gender was associated with lower odds of HF compared to males (OR = 0.45, 95% CI: 0.28–0.71, *p* = 0.001). Baseline eGFR showed a significant protective association (OR = 0.98, 95% CI: 0.97–0.99, *p* = 0.004). However, no significant associations were observed for age (OR = 1.02, 95% CI: 0.99–1.06, *p* = 0.178), duration of SGLT-2 inhibitor use (OR = 1.13, 95% CI: 0.89–1.44, *p* = 0.309), or baseline HbA1c levels (OR = 1.14, 95% CI: 0.99–1.31, *p* = 0.066). Time trends across years (2017–2020 compared to 2016) were not significantly associated with HF incidence (Table 3).

## 4. Discussion

The results of this study further support the significance of SGLT-2 inhibitors as a useful treatment choice in patients with T2DM, offering important insights into the preventive benefits of these medications in patients with T2DM at risk of heart failure.

The main outcome of our study is heart failure, a clinical syndrome defined by the heart’s incapacity to support venous return or meet the body’s metabolic needs [38]. Our results indicate that atherosclerosis and atherosclerotic cardiovascular diseases, such as acute myocardial infarction and stroke, are linked to a high risk of heart failure in patients with T2DM, which is consistent with the common precedents of HF, which include coronary artery disease, DM, and hypertension [39].

Peripheral artery disease is an important comorbidity in patients with HF, especially in patients with T2DM, and these closely related disorders can cause mutual complications [40]. Atherosclerosis, driven by chronic hyperglycemia, inflammation, and oxidative stress, underlies both peripheral artery disease and heart failure, as fatty plaques impair blood flow in peripheral and coronary arteries alike [41]. Endothelial dysfunction, a result of chronic hyperglycemia and systemic inflammation, further limits blood flow and oxygen delivery, worsening peripheral artery disease symptoms while also straining the heart and contributing to heart failure [42]. Also, in patients with T2DM, impaired angiogenesis disrupts tissue regeneration and blood vessel formation, accelerating the progression of peripheral artery disease and increasing susceptibility to ischemic events, infections, and delayed wound healing [43]. In line with these pathogenic pathways, our findings revealed a relationship between peripheral arterial disease and heart failure in individuals with T2DM.

Clinical indicators of renal function can be employed to assess CVD risk in patients with T2DM [44,45], because nephropathy is a significant risk factor for CVD in these patients [46]. Impairment to the kidneys frequently results in fluid overload and hypertension, which put further stress on the cardiovascular system and increases the risk of heart failure and CVD [47]. The risk of CVD is further increased by chronic hyperglycemia in T2DM, which encourages endothelial dysfunction, atherosclerosis, and artery constriction [48]. Additionally, anemia brought on by decreased erythropoietin production increases heart load, which can result in cardiac muscle hypertrophy and even heart failure [49]. Kidney dysfunction also interferes with lipid metabolism, causing dyslipidemia and accelerating the development of atherosclerotic plaque [50]. All of these interconnected factors combine to produce an endless loop in which nephropathy increases the risk for CVD in patients with T2DM while simultaneously indicating kidney dysfunction, and the findings of our investigation support this.

SGLT2 inhibitors are antidiabetic drugs that block glucose reabsorption in the proximal renal tubules, leading to glycosuria and improved glycemic control [51]. Their mechanism of action extends beyond lowering glucose levels, offering cardiorenal protection through multiple pathways. These include reduced intraglomerular pressure via restored tubuloglomerular feedback, decreased blood pressure, and improved vascular function [52]. SGLT2 inhibitors also reduce tubular workload and hypoxia, shift the metabolism towards ketogenesis, and enhance autophagy; additionally, they exhibit anti-inflammatory and antifibrotic effects, modulate mitochondrial function, and improve lipid metabolism [2].

SGLT2 inhibitors have demonstrated significant cardiovascular benefits in patients with T2DM; this is the main result of our current research. These drugs reduce glucose reabsorption in the kidneys, as mentioned above, leading to glucosuria and improved glycemic control [53]. However, the effect of SGLT-2i on reducing the risk of HF cannot be explained by its blood glucose-lowering action alone. The cardiovascular-protective effects of SGLT2 inhibitors are attributed to multiple mechanisms, including blood pressure reduction and improved insulin sensitivity [54].

At the cellular level, SGLT2 inhibitors help to lower inflammation, oxidative stress, and mitochondrial dysfunction in cardiac tissue [24]. SGLT2 inhibitors were shown to have anti-inflammatory properties that likely support their cardiovascular and renal benefits in individuals with T2DM. These agents activate AMP-activated protein kinase, which helps suppress pro-inflammatory pathways and decreases the production of inflammatory mediators. Additionally, they influence other inflammation-related pathways, such as the NLRP3 inflammasome and NF-κB activation [55]. SGLT-2i reduces oxidative stress, which is one of the major factors contributing to cardiovascular complications in patients with T2DM, by improving calcium signaling and reducing the production of reactive oxygen species [56]. SGLT2 inhibitors may directly bind to nutrient-deprivation sensors, promote mitochondrial health, and inhibit glucose transporter type 1, thereby improving cellular homeostasis and ATP production [57].

The cardioprotective effect of SGLT-2 inhibitors is also associated with their body weight-reducing effects, as this is a major prerequisite for T2DM and CVD. A meta-analysis found that monotherapy with SGLT-2 inhibitors was slightly better than a placebo in non-diabetic overweight or obese subjects [58]. This weight loss effect, although potentially moderated by counter-regulatory mechanisms, has been consistently noted in various studies [59]. Losing weight has been demonstrated to be essential in preventing heart failure in obese people [60]; therefore, SGLT-2i’s weight-loss effect may also lower the risk of heart failure for individuals with T2DM. The glucose-like peptide-1 (GLP-1) agonist has also been shown to lower the incidence of obesity-related heart failure and improve heart function by reducing body weight [61]. This supports the idea that SGLT-2 inhibitors’ ability to prevent HF could be more directly connected to their ability to lower body weight.

Clinical trials have shown that SGLT2 inhibitors decrease cardiovascular mortality, all-cause mortality, and hospitalization for heart failure in T2DM patients with established CVD [62]. These multifaceted actions make SGLT2 inhibitors a promising therapeutic option for CVD prevention in patients with T2DM, and our findings also suggest the same. However, as previously stated, randomized clinical trials and in vitro and in vivo laboratory studies have largely validated the cardioprotective effect of SGLT-2 inhibitors. Our study’s utilization of real-world data is crucial for assessing the long-term effectiveness of novel medications. While RCTs are the gold standard for assessing the safety and efficacy of novel interventions, their strict inclusion criteria often limit their generalizability [63]. Real-world data provide valuable information on the safety and effectiveness of medication in large, heterogeneous populations, reflecting everyday clinical practice [64]. Therefore, our study is considered significant because it uses real-world data to reveal the protective effect of SGLT-2 inhibitors against heart failure in patients with T2DM.

### Strengths and Limitations

This study leveraged real-world data from a well-documented T2DM population at the University Hospital of Debrecen, providing valuable information into clinical outcomes under routine healthcare conditions. The inclusion of diverse demographic, clinical, and laboratory variables enhances the comprehensiveness and robustness of the analysis. Our findings align with prior randomized controlled trials (RCTs), strengthening their external validity and supporting the generalizability of SGLT-2 inhibitors’ cardioprotective effects. The use of logistic regression, supported by AIC and BIC comparisons, ensures a methodological rigor suitable for the discrete nature of the dataset and the limited number of heart failure events. Adjusting for key clinical variables minimized potential confounding, while the grouping of SGLT-2 inhibitors allowed for a statistically robust evaluation of their overall impact.

Despite these strengths, the study’s single-center design may limit its generalizability beyond the Debrecen population. As an observational study, causal inferences are restricted, and unmeasured confounders, such as lifestyle and socioeconomic factors, may influence the results. The reliance on ICD-10 codes and clinical records introduces potential misclassification biases for comorbidities. The relatively short follow-up period may not fully capture the long-term effects of SGLT-2 inhibitors on heart failure outcomes. Additionally, medication adherence, a critical determinant of treatment effectiveness, was not accounted for in the dataset. These limitations highlight areas for future research to focus on to confirm and extend the findings.

## 5. Conclusions

SGLT-2 inhibitors appear to offer a protective effect against heart failure in patients with type 2 diabetes mellitus, independent of gender, age, and HbA1c levels, pointing towards the potential of SGLT-2 inhibitors in managing heart failure risk in this population. To the authors’ knowledge, this is the first time the cardioprotective effects of SGLT-2 inhibitors have been discussed using a clinical database focused on the Hungarian population. Further research should explore the underlying mechanisms driving this association and assess the long-term benefits across diverse demographic and clinical subgroups to refine treatment guidelines and optimize patient outcomes.

## Figures and Tables

**Table 1:** Baseline characteristics of patients with T2DM.

Variables		Baseline (%(*n*))
Age *		65.00 (58–71)
Gender	Female	54.72% (1391)
	Male	45.28% (1151)
Comorbidities (except for heart failure)	Atherosclerosis	28.21% (717)
	Myocardial ischemia	25.33% (644)
	Stroke	7.67% (195)
	Peripheral artery disease	15.11% (384)
	Nephropathy	17.15% (436)
	Retinopathy	14.63% (372)
	Neuropathy	18.69% (475)
Median HbA1c *		7.2 (6.5–8.2)
Medial LDL *		2.76 (2.05–3.50)
Median HDL *		1.20 (1.00–1.45)
Median triglyceride *		1.70 (1.24–2.50)

* Median (interquartile range).

**Table 2:** Comparison of patients with HF and those without HF, noting baseline characteristics.

Variable	Category	Without HF, N(%)	With HF, N(%)	*p*-Value
Gender	Female	1134 (98.52%)	17 (1.48%)	0.099
	Male	1380 (99.21%)	11 (0.79%)	
Age Group	<65	1252 (98.97%)	13 (1.03%)	0.723
	≥65	1262 (98.83%)	15 (1.17%)	
LDL Levels	<3.4 mmol/L	1530 (98.77%)	19 (1.23%)	0.642
	≥3.4 mmol/L	601 (99.01%)	6 (0.99%)	
**HbA1c**	**<7%**	**1057 (99.53%)**	**5 (0.47%)**	**0.01**
	**≥7%**	**1457 (98.45%)**	**23 (1.55%)**	
Triglycerides	<1.7 mmol/L	1104 (99.10%)	10 (0.90%)	0.383
	≥1.7 mmol/L	1243 (98.73%)	16 (1.27%)	
**Atherosclerosis**	**Yes**	**1812 (99.29%)**	**15 (2.09%)**	**0.003**
	**No**	**702 (97.91%)**	**13 (0.71%)**	
Stroke	Yes	2323 (98.98%)	4 (2.05%)	0.186
	No	191 (97.95%)	24 (1.02%)	
**AMI**	**Yes**	**1890 (99.58%)**	**20 (3.11%)**	**<0.001**
	**No**	**624 (96.89%)**	**8 (0.42%)**	
**Peripheral Artery Disease**	**Yes**	**2138 (99.07%)**	**8 (2.08%)**	**0.045**
	**No**	**376 (97.92%)**	**20 (0.93%)**	
**Nephropathy**	**Yes**	**2087 (99.10%)**	**9 (2.06%)**	**0.034**
	**No**	**427 (97.94%)**	**19 (0.90%)**	
**Retinopathy**	**Yes**	**2150 (99.08%)**	**8 (2.15%)**	**0.036**
	**No**	**364 (97.85%)**	**20 (0.92%)**	
Neuropathy	Yes	2048 (99.08%)	9 (1.89%)	0.066
	No	466 (98.11%)	19 (0.92%)	
**SGLT-2 Inhibitors**	**Yes**	**2268 (99.87%)**	**25 (9.23%)**	**<0.001**
	**No**	**246 (90.77%)**	**3 (0.13%)**	

Bold values indicate statistical significance (*p* < 0.05) based on Pearson’s chi-squared test.

**Table 3:** Multiple logistic regression model.

Variable	Odds Ratio [95% CI]	*p*-Value
**SGLT-2 inhibitor (yes/no)**	**0.55 [0.31–0.99]**	**0.046**
Age (years)	1.02 [0.99–1.06]	0.178
**Gender (female/male)**	**0.45 [0.28–0.71]**	**0.001**
Duration SGLT-2 inhibitor use	1.13 [0.89–1.44]	0.309
Baseline HbA1c (%)	1.14 [0.99–1.31]	0.066
**eGFR (mL/min/1.73 m^2^)**	**0.98 [0.97–0.99]**	**0.004**
year: 2017/2016	1.38 [0.59–3.22]	0.461
year: 2018/2016	0.85 [0.40–1.80]	0.671
year: 2019/2016	0.92 [0.49–1.74]	0.797
year: 2020/2016		

Bold values indicate statistical significance (*p* < 0.05) Odds ratios are adjusted for variables in the model.

## Data Availability

The datasets produced and/or analyzed in this study can be obtained from the corresponding author upon reasonable request.
